# Editorial: Metal Resistance in Microorganisms

**DOI:** 10.3389/fmicb.2022.899448

**Published:** 2022-04-11

**Authors:** Rob Van Houdt, Jon L. Hobman, Jean-Yves Matroule, Raymond J. Turner

**Affiliations:** ^1^Microbiology Unit, Belgian Nuclear Research Centre (SCK CEN), Mol, Belgium; ^2^School of Biosciences, University of Nottingham, Sutton Bonington Campus, Sutton Bonington, United Kingdom; ^3^Research Unit in Microorganisms Biology (URBM), Narilis Institute, University of Namur, Namur, Belgium; ^4^Department of Biological Sciences, Faculty of Science, University of Calgary, Calgary, Alberta, Canada

**Keywords:** metals, resistance, adaptation, microorganisms, bioremediation

The duality of metals puts them at the forefront of interest in microbial physiology. Whether metals have an essential biological role or not, at high concentrations they are extremely toxic as well as stable and recalcitrant. Nevertheless, modern technology is increasingly dependent on them. Excessive anthropogenic use generates continuous and increasing exposure to metals and metalloids. As a result, many environments are severely contaminated with metals. Although this creates serious environmental problems worldwide, the increasing demand for metals also drives efforts to meet this demand in a sustainable way as well as to increase the end-of-life recycling rate of them.

The interaction between microorganisms and metals is an area of active research. Microbes have co-evolved with the geological changes of the planet and have thus adapted to use metals in their biochemistry, but also have evolved to protect themselves against potential adverse interactions with metals. On the one hand, this teaches us how these interactions lead to adaptation and development of metal resistance, and on the other hand, how these processes can be exploited to remove or convert metals from contaminated environments, to recover them from waste streams, or to synthesize metal-based compounds using ecofriendly biological approaches. The interactions between microorganisms and metals have been studied at different levels. Often the first exploratory step is studying microbial communities in metal-contaminated environments, such as soil. These efforts broaden our knowledge of the diversity of bacterial species coping with metals, which could be exploited for bioremediation purposes (Yu et al.), and help to unravel the interactions between different community members in relation to metal tolerance and remediation (Lupini et al.).

Next, genomic insights, based on cultivable isolates or metagenomics approaches, mature the gene pool that is involved in metal resistance (Chen et al.; Huang et al.) as well as their genomic location and mobility, which will impact microbial adaptation (Huang et al.). In a subsequent step, the functional products of these genes and how these provide the ability to cope with metals can be scrutinized (Chen et al.; Rogiers et al.). Such data not only show how this is beneficial for bacteria in their specific niche, e.g., the survival of *Salmonella enterica* sv. Typhimurium in macrophages (Méndez et al.), but also how it can be employed for bio-based strategies for metal biomineralization, reduction and nanoparticle formation, as seen for tellurite and *Paenibacillus pabuli* (Farias et al.). The latter complements studies using cells and their metabolites, as evidenced by uranium phosphate biomineralization with *Penicillium simplicissimum* KS1 isolated from the flood water of a former uranium mine (Schaefer et al.), and silver nanoparticle formation by a *Viridibacillus* sp. soil isolate (Singh and Mijakovic), respectively.

Finally, the regulatory cascade involved in the regulation of these genes needs to be unraveled in order to fully characterize resistance and adaptation. Not only does this provide insights into how bacteria are primed to respond to metal stress (Carvalho et al.), it also provides mechanistic insights into regulation, for instance the negative regulation of the NmtA metallothionein from *Anabaena* sp. strain PCC 7120 by the α5 SmtB/ArsR metalloregulator AzuR (Divya and Acharya). Insights that are essential if such systems are further applied or exploited in applications.

The diversity of studies within this Research Topic illustrates the variety of questions and challenges that remain in the field of metal-microbe interactions. Here, we have achieved the goal of sampling the latest research on molecular mechanisms deployed by bacteria and communities to adapt and resist metals.

## Author Contributions

RV, JH, J-YM, and RT wrote sections of the manuscript. All authors contributed to manuscript revision, read, and approved the submitted version.

## Conflict of Interest

The authors declare that the research was conducted in the absence of any commercial or financial relationships that could be construed as a potential conflict of interest.

## Publisher's Note

All claims expressed in this article are solely those of the authors and do not necessarily represent those of their affiliated organizations, or those of the publisher, the editors and the reviewers. Any product that may be evaluated in this article, or claim that may be made by its manufacturer, is not guaranteed or endorsed by the publisher.

